# Challenges in exploring and manipulating the human skin microbiome

**DOI:** 10.1186/s40168-021-01062-5

**Published:** 2021-05-30

**Authors:** Manon Boxberger, Valérie Cenizo, Nadim Cassir, Bernard La Scola

**Affiliations:** 1 IRD, AP-HM, MEPHI, Aix Marseille Université, Marseille, France; 2 IHU-Méditerranée Infection, 19-21 Boulevard Jean Moulin, 13385 Marseille Cedex 05, France; 3 Groupe L’Occitane, R&D Department, Zone Industrielle Saint Maurice, 4100 Manosque, Alpes-de Haute-Provence France; 4 IRD, AP-HM, SSA, VITROME, Aix Marseille Université, Marseille, France

**Keywords:** Skin, Microbiome, Bacteria, 3D skin models, Probiotics, Cosmetics, Diseases, Culture, Next-generation sequencing

## Abstract

The skin is the exterior interface of the human body with the environment. Despite its harsh physical landscape, the skin is colonized by diverse commensal microbes. In this review, we discuss recent insights into skin microbial populations, including their composition and role in health and disease and their modulation by intrinsic and extrinsic factors, with a focus on the pathobiological basis of skin aging. We also describe the most recent tools for investigating the skin microbiota composition and microbe-skin relationships and perspectives regarding the challenges of skin microbiome manipulation.

Video abstract

**Supplementary Information:**

The online version contains supplementary material available at 10.1186/s40168-021-01062-5.

## Introduction

The skin is the largest organ and the outermost interface between the human body and its environment. For decades, the skin surface was estimated to have an area as high as 2 m^2^, but recently, by taking into account the appendages, the estimate has increased to as high as 25 m^2^ [1]. Many regional differences overlap in the skin topography. For instance, temperature and humidity are higher at vaulted sites, such as the groin or armpit (approaching 37°C, the body’s core temperature) and lower at the body’s extremities (fingers and toes, approximately 30°C). Sebaceous gland density is an important variable factor involved in the secretion of many lipidic compounds, including fatty acids, that contribute to the acidification of the skin pH, which varies between 4.2 and 7.9 depending on the site of measurement [2].

These characteristics induce many possibilities for creating different ecological niches housing numerous commensal bacteria as well as fungi, viruses, archaea, and mites [3] in a network that varies in terms of its density and composition. Altogether, these microorganisms define the skin microbiota. According to Grice et al. [4], skin microbiota diversity may be higher than gut microbiota diversity. The vulnerability of this microbe network lies in the many intrinsic and extrinsic factors that affect it. The implications for wound healing and protection against potential pathogens or environmental conditions highlight a crucial role of skin homeostasis. Indeed, recent studies have identified associations between shifts in these commensal populations and physiological changes, such as aging and diverse dermatological diseases, not only in humans but in all vertebrates [5, 6].

With the recent advent of molecular biology and next-generation sequencing (NGS) as tools for microbiological identification, knowledge about the skin microbiota has grown exponentially. However, culture methods remain an essential tool for studying the characteristics of microorganisms in vivo. The manipulation of the skin microbiota represents a considerable challenge in clinical and cosmetic practice. This review discusses recent findings regarding the skin microbiome and its role in human health, aging, and disease.

## Skin microbiota composition

### Bacteria

Byrd et al. [3] reported the top ten bacterial species found on the skin through site area surveys in healthy volunteers based on high-throughput gene sequencing analysis. Human skin samples were found to be dominated by gram-positive bacteria belonging to the genera *Staphylococcus* spp., *Corynebacterium* spp., *Enhydrobacter* spp., *Micrococcus* spp., *Cutibacterium* spp., and *Veillonella* spp. A culture-based study by Myles et al. [7] that focused on the culturable fraction of gram-negative bacteria (GNB) from the human skin identified *Roseomonas mucosa*, *Pseudomonas* spp., *Acinetobacter* spp., *Pantoea septica*, and *Moraxella osloensis* as commensal residents. Other studies have verified that gram-negative bacteria (GNB), including *Enterobacteriaceae*, nonfermenting GNB, and anaerobes, are underestimated skin commensal organisms but are also part of the transient fraction of the skin microbiota [8, 9].

### Candidate phyla

To date, few studies have reported the existence of TM7x (*Saccharibacteria* spp.) [10, 11] and TM6 sequences from skin samples [12]. These “candidate phyla” are considered to be not-yet-culturable bacteria and constitute a field of growing interest because such organisms are expected to be linked to pathologies such as periodontitis [13].

### Archaea

As part of the Human Microbiome Project, Moissl-Eichinger et al. [14] focused their research on the detection of overlooked Archaea on human skin by analyzing 13 samples collected from healthy torso skin. *Thaumarchaeota* and *Euryarchaeota* were shown to be carried by all the human subjects analyzed [14, 15]. Phylogenetic analysis of *Thaumarchaeota* placed them close to ammonia-oxidizing archaea from the soil. Moreover, although it remains to be proven, the role of these archaea could be explained by chemolithotroph ammonia turnover, which could influence the pH regulation of the human skin and therefore the natural protective barrier of the body [15, 16].

### Viruses

Within the viral fraction found on the skin, bacteriophages are predominant. The lytic activity of bacteriophages has been linked to the modulation of bacterial populations, and thus, bacteriophages participate in the homeostasis of the skin microbiota. Through culture-based approaches and genomic analysis of skin samples, Liu et al. [17] revealed an increased frequency of *C. acnes* phages isolated from healthy individuals compared to patients with *Acne vulgaris* and suggested that phages may play a role in modulating skin bacterial populations. Metagenomic shotgun sequencing analysis suggests that *Cutibacterium* and *Staphylococcus* phages are the most abundant skin phages, while other phages, such as *Streptococcus* and *Corynebacterium* phages, are also present but at lower relative abundances [18]. Byrd et al. [3] reported the top ten viruses found on the skin. Phages were identified as well as *Acheta domestica*; *Densovirus*; *Alphapapillomavirus*; *Human papillomavirus* (β), (γ) and (μ); *Merkel cell polyomavirus*; *Molluscum contagiosum virus*; *Polyomavirus* HPyV7; *Polyomavirus*; *HpyV6 RD114* retrovirus; and Simian virus. *Papillomaviruses* and *Molluscum contagiosum* are known to cause dermatological lesions, such as warts. *Merkel cell polyomavirus* is implicated in the development of carcinoma. The question of the underappreciated abundance of phages was discussed recently by Hannigan et al. [19]. Whether the presence of these phages plays a role in skin microbiota dysbiosis or the expression of virulence or antibacterial genes needs to be further studied.

### Eukaryota: fungi and demodex

To date, fungi, including *Malassezia*, *Cryptococcus*, *Rhodotorula*, and *Candida* species, have been identified as human skin commensal organisms. Culture-based studies have identified *Malassezia* spp. as the main genus of commensal skin fungi*.* The fungal community composition, unlike the bacterial fraction, was previously considered to be similar over all body areas [3]. However, recent studies showed that *Malassezia* spp. predominated at the central sites of the body and arms, while foot sites were colonized by a more diverse combination of fungi. *Demodex* are mites of the family *Demodicidae* and live in seborrheic areas of the skin, such as the face and hair [3, 18, 20]. *Demodex* are also widely found on the eyelids and the nasal ala. Two species have been identified from human samples: *Demodex folliculorum* and *Demodex brevis* [21], but such organisms remain difficult to breed. Notably, the microbiota of these organisms has been studied to increase our knowledge about how they are linked with cutaneous diseases, such as papulopustular rosacea [22].

## Role of the skin microbiome

### The maintenance of skin homeostasis plays a protective role against potential pathogens and environmental issues

The skin microbiome contributes to the barrier function of the skin and ensures skin homeostasis. The secretion of protease enzymes by skin microbes is involved in the desquamation process and *stratum corneum* renewal. Sebum and free fatty acid production are involved in pH regulation [23]. The secretion of lipase enzymes is involved in lipidic film surface breakdown. In addition, urease enzymes are implicated in urea degradation. Other roles of the microbiota include the production of biofilms, bacteriocins, and quorum sensing [24, 25]. Moreover, the skin microbiota plays an important role in protecting against potential pathogenic microorganisms by competition [26, 27] and antimicrobial peptide (AMP) production by commensal bacteria [28, 29] or *Malassezia* fungi, which produce a range of indoles that inhibit many other yeasts and molds [30].

### Training and communication with the immune system (Fig. 1)

Skin commensal bacteria have a close relationship with host immune cells from the beginning of their life, and skin resident T cells are thus trained to respond to potential transitory pathogenic bacteria [20, 31]. Meisel et al. [23] showed that the expression of 2820 genes was modulated in mice in response to microbial colonization. A notable proportion of these genes was related to the host immune response and showed roles in processes such as cytokine production, the complement cascade, and the signaling and homing of T cells. A specific strain of *Staphylococcus epidermidis* was shown to be able to produce 6-*N*-hydroxyaminopurine, which may confer protection against skin cancer [32].

**Fig. 1:**
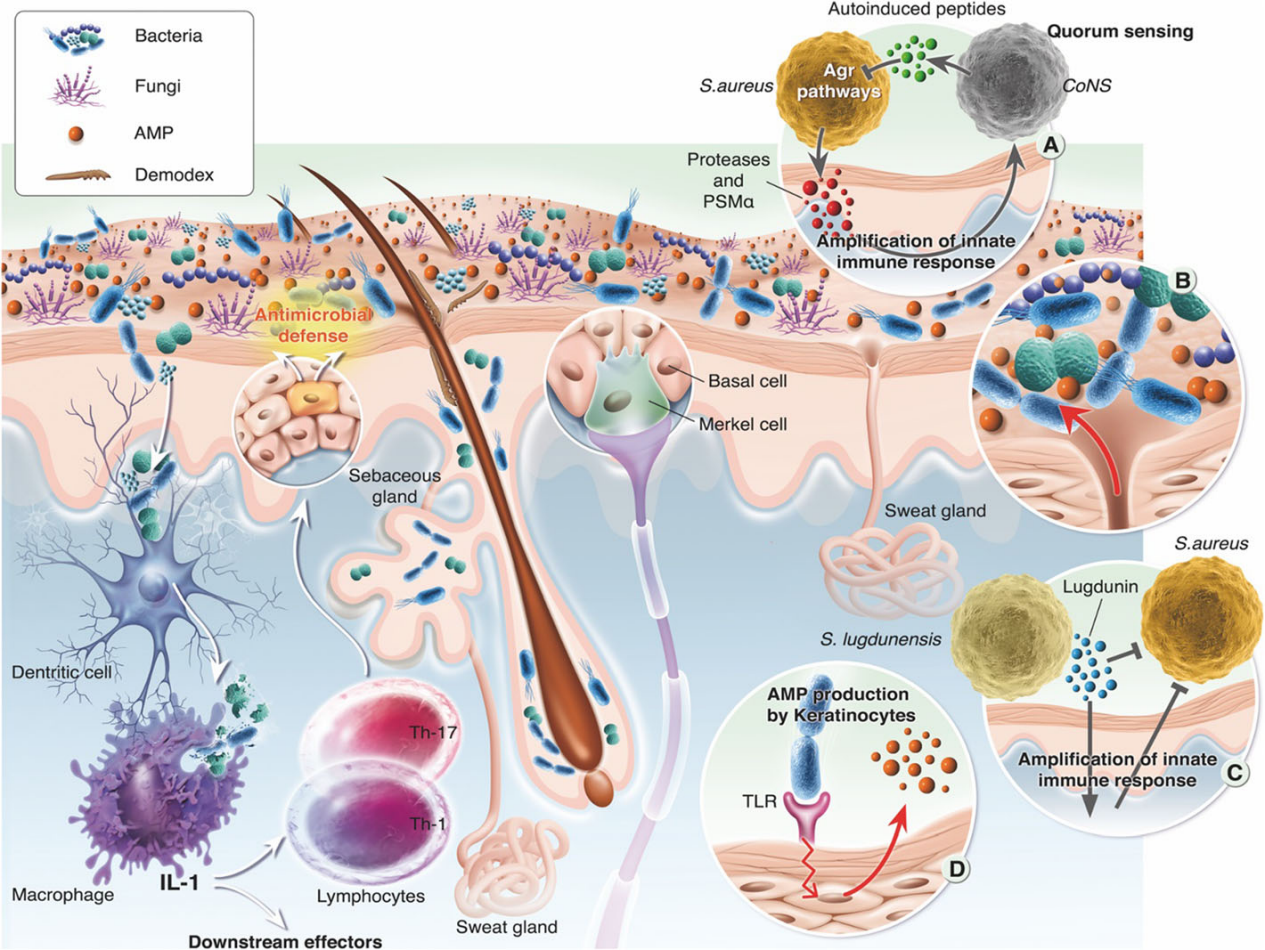
Skin microbiota, its roles, and its relationship with the immune system. The skin microbiota is composed of bacteria, fungi, archaea, viruses, and mites (*Demodex*) that are related to the immune system through dialog with resident dendritic cells resulting from complement activation. **a** The immune system is enhanced by the quorum-sensing process between bacterial populations, which can limit the overgrowth of potential pathogens, or by the production of certain antibiotics, such as lugdunin (**c**). Microbiotic homeostasis is dependent on the production of antimicrobial peptides (AMPs) both by bacteria themselves and by host cells, such as keratinocytes and sebocytes (**b** and **d**).

### Wound repair

As described above, skin commensal organisms are in constant crosstalk with the immune system and are thus also involved in wound healing. Leonel et al. [33] synthetized the current knowledge of this topic, which appears to be conflicting. For example, the absence of commensal skin microorganisms has been shown to have a positive effect on wound closure during healing [34]. On the other hand, in another study, the presence of *Staphylococcus epidermidis* was noted to be a positive factor related to unconventional repair mechanisms specific to commensal bacteria via the recruitment of regulatory CD8 T cells [35]. This finding is consistent with the beneficial skin microbiota effect noted by Lai et al. [36] and the negative effect of skin microbiota dysbiosis [37, 38]. Future investigations are needed to elucidate the influence of the skin microbiota in this process, given the complexity of its definition and its heterogeneity.

## The skin microbiota composition depends on many factors (Fig. 2)

### Intrinsic factors

#### Skin site, “biogeography” factor

Grice et al. [4] analyzed 20 different skin sites in 10 healthy humans. They found that *Propionibacteria* species and *Staphylococci* species predominated at sebaceous sites, and *Corynebacteria* species predominated at moist sites, although *Staphylococci* species were also represented. A mixed population of bacteria resided at dry sites, with a greater prevalence of *β-Proteobacteria* and *Flavobacteriales*.

**Fig. 2:**
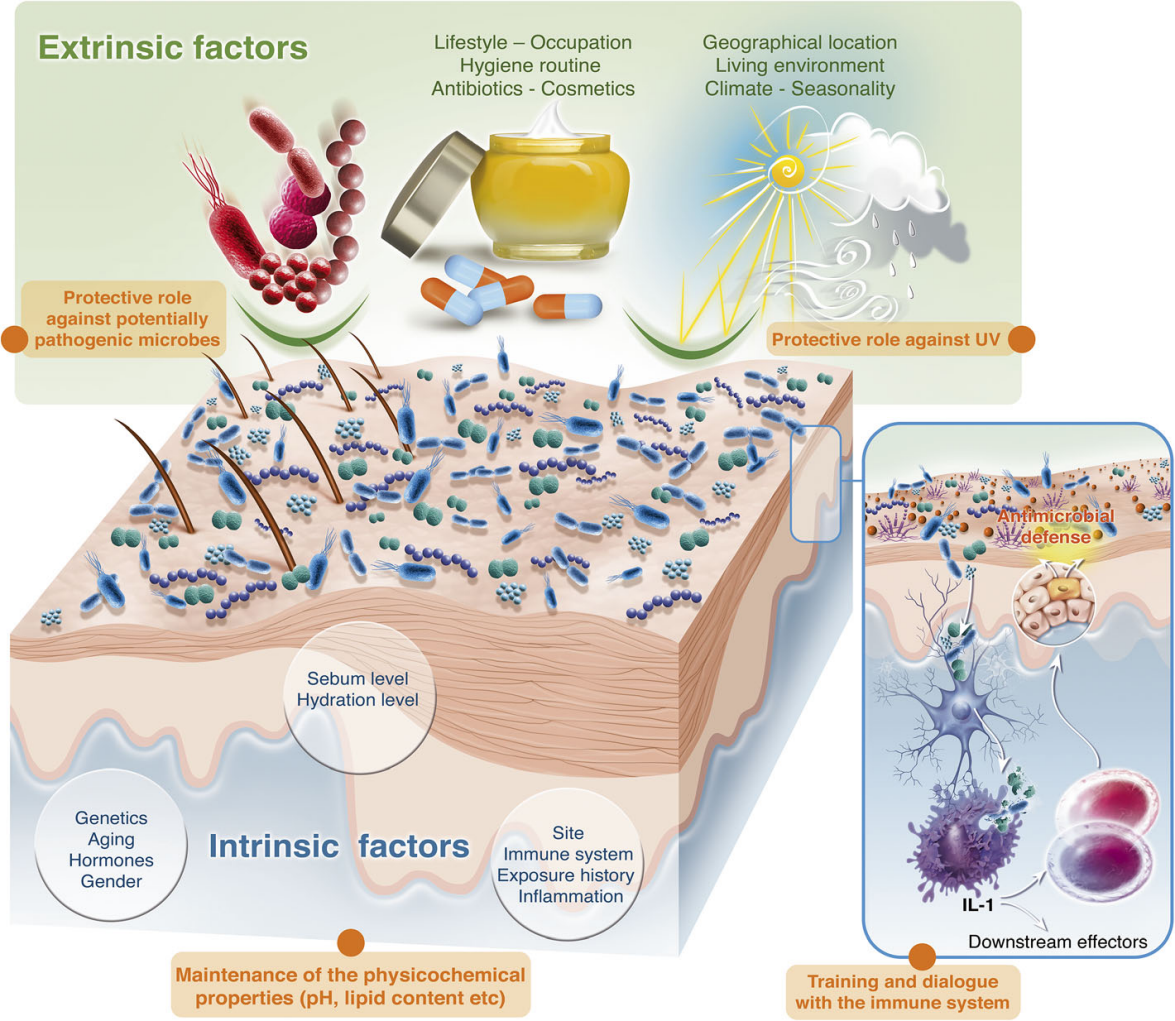
Factors influencing the composition and function of the human skin microbiota and its function. The skin microbiota is shaped by numerous factors: extrinsic (such as lifestyle that embodies occupation, hygiene routine, use of drugs and cosmetics) and intrinsic (genetics, aging, sex, site of the body, etc.) These factors influence the roles of the skin microbiota, implying protection against potential pathogens or climate perturbation as well as the maintenance of skin integrity.

#### Intra- and interpersonal variability

Costello et al. showed that the phylogenetic diversity of different skin sites was greater than that of communities in the gut, the external auditory canal, or the oral cavity [39]. Interpersonal variation was found to be greater than intrapersonal variation over time. More recently, these observations have been supported by Perez et al. [40], who showed that the arms present significantly less intragroup variation than the axilla or the scalp, and the axilla exhibits the greatest intragroup variation.

#### Ethnicity

Ethnicity has been shown to contribute to skin microbiome variation and is partly linked to lifestyle. Indeed, Harker et al. [41] reported the differences in the axillary microbiota, such as significantly lower abundances of *Staphylococcus* species and greater abundances of *Corynebacterium* species, linked to different genotypes of the gene ABCC11. Between A/A and A/G or G/G individuals, dimorphism was observed between East Asian people and European or African people. Leung et al. [42] indicated that the microbial composition of Chinese people was different from that of other ethnic groups through analysis of metagenomics data from different studies analyzing the palms of hands. Perez et al. [40] showed that the arm microbiota of African American men was relatively homogenous and significantly different from those of all other groups, including the African-continental group. Similarly, the axillary microbiota of East Asian men was highly homogenous and significantly differed from that of other groups. Li et al. [43] found that a unique microbial composition was associated with East Asians compared to Caucasians and Hispanics. East Asians presented higher levels of total bacteria and proteobacteria than the other groups. The *Corynebacterium* species distribution was analyzed, and *Corynebacterium variabile* was found to be present exclusively in Hispanics, while *Corynebacterium kroppenstedtii* was only detected in the East Asian group. However, the definition of ethnicity in the era of globalization with permanent migratory crossings remains elusive.

#### Gender

Physiological differences between male and female skin environments, such as differences in hormone metabolism, the perspiration rate, and skin surface pH, have been described. Fierer et al. [44] found significant differences between men and women in terms of alpha diversity, but these differences seemed to be related to specific sites and to affect specific age groups. The hand microbiota of women was characterized by greater alpha diversity than that of men, but no specific taxa were found. In terms of relative abundance, they showed that *Cutibacterium* and *Corynebacterium* were more abundant in men than in women. In women, *Enterobacteriales*, *Moraxellaceae*, *Lactobacillaceae*, and *Pseudomonadaceae* were more abundant than in men. Oh et al. [45] observed no sex-associated differences through the study of males and females between the ages of 2 and 40 years regardless of their age based on swabs of the antecubital and popliteal fossae, the volar forearm, and the nares. By studying the axillary vault, Callewaert et al. [46] found that two main groups could be distinguished by characterizing whether the predominantly colonizing genus was *Staphylococcus* or *Corynebacterium*. Females predominantly clustered within the *Staphylococcus* cluster, whereas males clustered more frequently with the *Corynebacterium* cluster. Prohic et al. [47] did not find a significant influence of sex by studying the distribution of *Malassezia* species, whereas a significant impact on archaeal diversity and the archaeal community composition was observed [14]. Leung et al. [48] showed that males were characterized by higher abundances of *Cutibacterium*, *Staphylococcus*, and *Enhydrobacter*, whereas *Streptococcus* was more abundant in the female population. Jo et al. [49] suggested that sex may affect the mycobiome structure during sexual maturation. The *Epicoccum* and *Cryptococcus* genera were found at sebaceous sites in males, whereas *Malassezia* was enriched in females. Zhai et al. [50] showed that males presented greater species richness than females, but the sex differences in the community structure were only present in certain age groups and at particular skin sites. These differences could be found on the upper back of adolescents and elderly people and the cheeks of young adults. Li et al. [43] observed that males presented greater amounts of *Corynebacteria* than females, though this difference was not significant. Moreover, males exclusively hosted *Corynebacterium amycolatum* and *Corynebacterium kroppenstedtii*, in contrast to females, who hosted only *Corynebacterium urealyticum* and *Corynebacterium variabile*. By conducting a study based on cultured bacteria, Shami et al. [51] found no significant effect of sex on the number of bacteria isolated from four groups (young people, elderly people, males, and females).

#### Aging

Although it is known that the skin microbiome is relatively stable over time in the medium term [18], aging is known to be one of the main factors influencing the skin microbiota composition. In 2019, Dimitriu et al. [52] sampled 495 people of various origins at four skin sites and the mouth and considered aging to be the fourth most important factor affecting skin microbiome variation following lifestyle, physiology, demographic proprieties, and pigmentation.

**Fig. 3:**
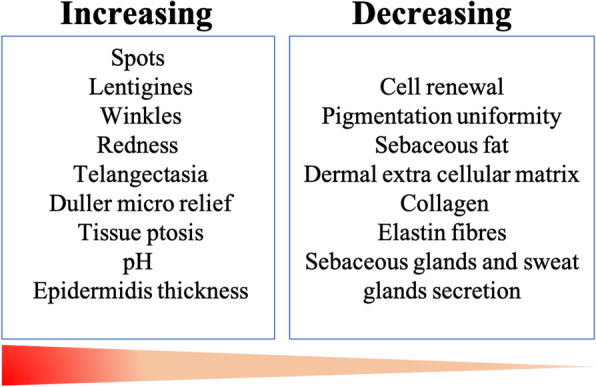
Skin features associated with aging

Indeed, aging is associated with many shifts in skin features, such as spot and wrinkle appearance, modified sebaceous gland activity, and dermal compound production [53] (Fig. 3), which impact the previously established ecological conditions for cutaneous microorganisms.

Several studies have described higher bacterial alpha diversity in skin samples in elderly people. Somboonna et al. [54] studied the skin bacterial composition of 30 healthy Thai females aged 19 to 57 years at the same sites and found that *Planctomycetes* and *Nitrospirae* were more prevalent in the teenage group. A Japanese cohort was analyzed in 2017 by Shibagaki et al. [55], who revealed 38 different bacterial species, including many oral bacteria, which significantly differed between the two age groups with skin site dependency. They showed a reduction in the relative abundance of the dominant skin genus *Cutibacterium* in the cheek, forearm, and forehead microbiota of older adults; an increased proportion of *Corynebacterium* in the older group on the cheek and forehead; and an increased proportion of *Acinetobacter* on the scalp in the older group. In another study, Jugé et al. [56] analyzed the forehead skin microbiota of 34 healthy Western European women. They observed an increase in *Proteobacteria* and a decrease in *Actinobacteria* populations on older skin. Within the latter phylum, there was a significant increase in the relative abundance of *Corynebacterium* and a decrease in the relative abundance of *Cutibacterium*. These data are consistent with Shibagaki et al. [55], who described the previously identified phenomenon of decreased production of sebum associated with aging, which could induce a loss of nutrients available for such bacteria and induce a spread of opportunists. Dimitriu et al. [52] also demonstrated that two mutually exclusive *Corynebacterium* OTUs could be correlated with skin aging. Indeed, a *Corynebacterium* OTU with a similar trend to *Corynebacterium kroppenstedti* displaced another *Corynebacterium* OTU. In 2014, Prohic et al. [47] collected samples from the trunk and scalp of 100 people by scraping and observed variation in *Malassezia* species with age. *Malassezia furfur* was characteristic of the trunk skin of children, whereas *Malassezia restricta* was predominant on the scalp of 21- to 35-year-old individuals. The *Malasseiza* population was more prominent in the 36- to 50-year-old group, and *M. sympodialis* was the predominant species on the trunk skin in older subjects.

Among less-studied skin commensal microorganisms, such as *Demodex*, Jacob et al. [21] showed that the prevalence of *Demodex* mites on the skin was positively correlated with aging and reached up to 95% in people over 71 years old. For *Archaea*, Moissl-Eichinger et al. [14] showed a greater abundance in human subjects older than 60 years compared to middle-aged human subjects.

### Extrinsic factors

#### Mode of delivery

In newborns, bacterial skin colonization has been shown to be influenced by the mode of delivery, the postpartum environment, and the influence of medical staff [57–59]. This primary microbiota is transitory and largely influenced by environmental factors and subsequently evolves towards a microbiota that is close to that of adult skin, particularly to the microbiota of hydrated zones. From the age of 3 months, regionalization of the skin microbiota of the child is observed.

#### Lifestyle, hygiene routine, and cosmetics

Differences are observed in the skin microbiome of people living in different environments (rural or urban) [60–62], which are also correlated with the presence of domestic animals [63]. In contrast, the microbiota of people who live together tends to converge, even if they are not genetically related or intimate with each other [64]. Staudinger et al. [65] showed that the use of makeup, including foundation and powder, significantly increased community diversity on the forehead skin. The beneficial effects of some cosmetic compounds, such as preservatives, against the growth and biofilm formation of cutaneous *S. aureus* or pathogenic *C. acnes* have been described [66]. These chemical compounds are also involved in the inhibition of commensal bacteria survival [67]. Emulsifiers have been shown to favor the growth of potential pathogens, such as *S. aureus* [68]. Other studies have revealed the modulation of the diversity of archaeal and bacterial populations, and chemical skin compounds are correlated with changes in the hygiene/cosmetic routine (use of deodorant, moisturizer, or “historical” soap formulated with potash). Such shifts could result in an increased nutrient supply from these products [69–72]. These results led to the filing of numerous patents aimed at stabilizing or enriching the skin microbiota with beneficial bacteria (e.g., Jessica Wilson, “Personal cleansing compositions and methods of stabilizing the microbiome” 2016 Patent US20190053993A1; Greg Hillebrand, “Method and topical composition for modification of a skin microbiome” 2013 Patent EP3049533A1).

#### Antibiotics

Zhang et al. [37] demonstrated that the oral intake of vancomycin decreases the bacterial density and alters the bacterial composition in skin wounds, which may contribute to delayed wound repair in mice. In accordance with these data, SanMiguel et al. [73] showed that topical antibiotics can alter the resident skin bacteria for several days and implicated a decrease in the commensal *Staphylococcus* spp. population, which is known to compete for colonization with pathogenic *Staphylococcus aureus*. Recently, Park and Lee [74] found that oral administration of doxycycline was linked to a decrease in *Cutibacterium acnes* relative abundance, an increase in *Cutibacterium granulosum*, and thus an improvement in the clinical signs in 20 acneic patients, highlighting the implications of antibiotic use for the modulation of the skin microbiota.

#### Geographical location, climate, and seasonality

A recent study showed a greater benefit of an alpine climate compared to a maritime climate, which differ in pollution and UV radiation levels, for the treatment of atopic dermatitis in children [75]. This observation needs to be confirmed in subjects with healthy skin [76]. Other authors showed that after seawater exposure, exogenous bacteria were present on the skin for at least 24 h after swimming and that ocean water exposure removed normal resident bacteria from the human skin. Likewise, elevation, which is related to extreme environmental conditions, has been shown to disturb skin microbiome stability [77]. Moreover, airborne pollution [78] has been shown to degrade skin microbial population diversity.

## Diseases

Table 1 synthetizes the identified associations with commensal community dysregulation related to dermatological pathologies, which encompass widespread conditions, such as acne vulgaris and seborrheic or atopic dermatitis, and less common conditions, such as vitiligo or lupus erythematosus.

**Table 1:** Dermatological pathologies associated with the modification of the skin microbiota

Pathology	Microorganism correlated with pathology	References
Acne vulgaris	Shifted microbial composition implying *Cutibacterium acnes*.	Platsidaki et al. [79]; O’Neil and Gallo [80]
Atopic dermatitis	*Malassezia*.	Hiruma et al. [81]
	Increased *Staphylococcus aureus* and reduced quantities of *Cutibacterium acnes* and *Lawsonella clevelandensis*.	Francuzik et al. [82]
Seborrheic dermatitis	*Acinetobacter*, *Staphylococcus*, and *Streptococcus* predominated at lesioned sites.	Tanaka et al. [83]
Pityriasis versicolor	*Malassezia* spp.	Prohic et al. [47]; Moallaei et al. [84]
Blepharitis Chalazion Pterygium	*Demodex*.	Tarkowski et al. [85]
Papulopustular rosacea	Increasing population of *Demodex* mites.	Murillo et al. [22]
	*Demodex* microbiota.	
	*Proteobacteria* and *Firmicutes* population increased and *Actinobacteria* population decreased.	
Psoriasis	Depending on sampling method and sites.	Visser et al. [86]; Chang et al. [87]; Stehlikova et al. [88]
	Swabs and biopsy samples from psoriatic lesions were enriched in *Firmicutes*.	
	Increased abundance of the genus *Streptococcus* and a low representation of *Cutibacterium*, while presenting discordant results on the representation of *Staphylococcus*.	
	Swabs from psoriatic lesions on the back and the elbow show increased abundance of *Brevibacterium* spp. and *Kocuria palustris* and *Gordonia* spp.	
	Significantly higher abundance of the fungus *Malassezia restricta* on the back and sympodialis on the elbow.	
	Occurrence of *Kocuria*, *Lactobacillus*, and *Streptococcus* with *Saccharomyces*.	
	*Staphylococcus aureus* found to be more abundant in both psoriatic nonlesional and lesional skin while *Staphylococcus epidermidis*, *Cutibacterium acn*es, and *Cutibacterium granulosum* were more abundant in healthy skin. Incidence on the polarization of the Th17.	
Vitiligo	Decreasing diversity and lower association between microbial communities in affected sites.	Ganju et al. [89]
Skin cancers	Production of AhR ligand by *Malassezia* spp.	Gaitanis et al. [90]
	Skin bacterial load and AMP expression.	Natsuga et al. [91]
Actinic keratosis Cutaneous squamous cell carcinoma	*Propionibacterium* and *Malassezia* at higher relative abundances in healthy tissues. *Staphylococcus aureus in* relatively more abundant in lesional tissues.	Wood et al. [92]
Diabetic foot ulcer	Decreasing population of *Staphylococcus* species, increased population of *S. aureus*, increased bacterial population	Redel et al. [93]
Lupus erythematosus	Decreased abundance and uniformity of the microbial populations. *Staphylococcus epidermidi*s through the *Staphylococcus aureus* infection pathway.	Huang et al. [94]

## How to investigate the skin microbiota

### Sampling method

The uniqueness of skin characteristics makes it necessary to standardize and validate the methods used in microbiome research, which would allow comparisons between different studies [27]. The first parameter to keep in mind is the study design, which comprises the strict screening of the subjects involved in the study and the collection of all information that could influence the microbiome variation. The second parameter is sample processing. Samples obtained from swabbing, scraping, and tape strips provide information on the superficial microbiota composition, whereas biopsies offer the opportunity to study microorganisms that could inhabit the deepest layers of the skin [95]. Recently, Ogai et al. [96] completed a comparison between these sampling methods and showed no difference in the results of studying the microbiota by using swabs or tape strips for NGS analysis, but tape strip sampling was shown to be superior when the results were obtained by culture. Verbanic et al. [97] noted the necessity of improving the preparation of samples obtained via the swabbing method so that a sufficient DNA yield is obtained for 100% of samples (the percentage is 25% with traditional preparation). Pedrosa et al. [98] noted that direct plate contact with the skin for the recovery of *Malassezia* species was more convenient than the tape stripping method.

### Transport and storage conditions

Kong et al. [27] noted that immediate freezing at −80°C after sampling was generally preferred and that freeze-thaw cycles must be avoided. The optimization of sample collection and processing through the use of a protectant medium (transport medium that conserves viability and prevents the growth of microorganisms) and the control of storage temperature and time have a considerable effect on the results obtained by analyzing stool samples [99, 100]. Such conclusions remain to be assessed specifically in relation to the improvement of skin microbiota analysis.

## Microorganism identification methods: culture vs*.* nonculture tools

The culture of microorganisms is a historical method for studying their characteristics and properties. With recent advances in molecular biology, this fundamental tool has been shelved in favor of next-generation sequencing methods, which are more sensitive and faster than culture. However, next-generation sequencing does not provide all the information needed to understand the habits of microorganisms in vivo; for example, it provides no information about the viability of the detected organisms [98]. A goal that is as important as the improvement of sampling and storage methods is the improvement of culture parameters in efforts aimed at isolating the viable and culturable fraction of the skin microbiome, which presents its own particularities and shows certain consistent traits [64]. For example, Myles et al. [7] showed that when using a low-nutrient culture medium (R2A), inhibition of the gram-positive fraction by treating the sample with vancomycin and a reduced incubation temperature led to the isolation of the gram-negative fraction of the skin microbiota. Moreover, other parameters of the protocol could be adjusted to obtain more efficient culture media for the growth of diverse skin microorganisms and to improve the methods of colony identification [101, 102]. In these efforts, the culturomics method was improved by Lagier et al. [103], which allowed the discovery of multiple unknown bacteria. By using these methods (i.e., the combination of multiple culture media and conditions), Timm et al. [104] collected more than 800 strains, including more than 30 bacterial genera and 14 fungal genera. However, because this technique requires fastidious and time-consuming work, an increasing number of scientific teams have reinstated this method uniquely or with the use of complementary metagenomic tools [30, 105, 106].

The democratization of metagenomic technologies has induced a shift in interest related to human-associated microorganisms. The skin microbiota has been largely underestimated in terms of diversity, which has persisted because of culture techniques that induce bias due to the growth of microbes in artificial settings [106]. To apply this kind of method for skin microbiome analyses, particular attention is needed at each step of the protocol, including the DNA extraction method, library construction, sequencing step (e.g., primer selection, the chosen platform [88], and the use of blanks and controls), and subsequent analysis (e.g., the selected database and software) [27, 106–108]. Furthermore, advanced methods to isolate and cultivate difficult strains by reverse genomics have been recently proposed [109].

## Future insights

### Study of the microbe-skin relationship: the development of 3D skin models

Many biological models have been produced in an attempt to reconstitute the skin-microbiome interaction with different complexity levels. The first studies consisted of culturing human skin cells, mainly keratinocytes or sebocytes, with bacteria or their metabolites. The main goal of these studies was to understand the pathways involved in pathogen infections or commensal benefits for the skin. Keratinocytes incubated with sterile filtered *Staphylococcus aureus* medium showed increased production of proteolytic enzymes, followed by the degradation of skin barrier proteins, such as desmoglein-1 and filaggrin [110]. In contrast, some metabolites produced by *S. epidermidis* could increase the keratinocyte production of antimicrobial peptides via Toll-like receptor 2 activation [36]**.**

Using living bacteria, several studies showed that immediately after inoculation, different *Staphylococcus s*trains showed an increase in epidermal tight junctions (TJs) [111, 112]. However, after several hours of colonization, *S. aureus* decreased the number of TJs and, subsequently, that of two other types of epidermal junctions, adherent junctions (AJs) and desmosomes, whereas under the same conditions, *S. epidermidis* showed a minor effect. Other studies focused on cell viability and inflammation revealed that pathogenic strains such as *S. aureus* or *C. acnes* or their metabolites induced cell cytotoxicity and increased the production of pro-inflammatory cytokines in skin cells [113, 114].

Skin commensal organisms have also been incubated with skin metabolites to mimic the crosstalk between skin cells and the skin microbiota. As the largest neuroendocrine organ of the human body, the skin produces neurotransmitters, especially stress mediators, including catecholamines or substance P. *S. epidermidis*, *S. aureus*, and *C. acnes* can detect these molecules via specific receptors and respond with increased biofilm formation or production of toxins, resulting in a more virulent phenotype that causes more skin cell damage [115, 116].

Polymicrobial biofilms formed by a mixture of commensal strains (*Staphylococcus epidermidis* and *Micrococcus luteus*) and pathogens (*Staphylococcus aureus* and *Pseudomonas aeruginosa*) were also used to study the interactions among commensal organisms, pathogens, and human keratinocytes. The authors observed that the commensal organisms reduced the damage caused to the keratinocyte monolayer by pathogens, reduced biofilm thickness, and formed a layer between the keratinocytes and pathogens [117]**.**

Due to the faster growth rate of bacteria than human cells, 2D models cannot be maintained for more than 24 h. Moreover, cells cultivated in monolayers do not reflect the skin surface. These cells are more reflective of the conditions in wounds or defective skin barriers. These limitations have led to the development of more complex models that better reproduce the skin barrier with its dry environment to study long-term interactions between the skin and its microbiota.

Thus, 3D skin models have been colonized with bacteria. These models are now widely used for dermatological and cosmeceutical studies. The simplest model, the reconstructed human epidermis (RHE), is composed of primary human keratinocytes grown on a decellularized dermis or a porous membrane. Air-liquid culture allows the formation of a fully differentiated epidermis and the formation of a functional barrier. When a living dermis is also present, the model is referred to as full-thickness skin (FT-skin).

Many studies have used these models to study the bacterial, fungal, or yeast infection process. Using an RHE model, Lerebour et al. [118] described differential adhesion properties between *S. aureus*, a hydrophilic strain, and *S. epidermidis*, a relatively hydrophobic strain, suggesting that rendering the skin surface more hydrophobic would restrict microbial adhesion. When the epidermis is locally removed, either by punching or thermal injury, these models can reproduce a skin wound environment by making the dermis accessible to bacteria. These models have been widely used to study the infection process by following microbial growth and tissue damage [119–126]. These infection models have mostly been used to identify potential antibacterial treatments. For example, when a biofilm-forming *S. aureus* was inoculated at high density (i.e., >10^7^ CFU) onto a 3-mm-punched FT-skin model, plasma treatment reduced the number of adherent colonies after 24 h [119]. In another study, wound infection with methicillin-resistant *S. aureus* (MRSA) after thermal injury in an FT-skin model revealed significant growth of MRSA after 24–48 h. Skin exposure to MRSA increased the expression of inflammatory mediators, such as TLR2, IL-6, and IL-8, and the antimicrobial proteins human β-defensin-2, human β-defensin-3, and RNAse7. Moreover, locally applied mupirocin effectively reduced MRSA counts in a thermal wounded skin model by more than 99.9% within 24 h [120].

More recently, with the emerging role of the skin microbiome in skin health, 3D models have been colonized with commensal bacteria in the absence of any stress or injury. Unique commensal bacterial strains, among which *S. epidermidis* and *C. acnes* are the most represented, have been inoculated and cultivated for up to 4 days on the skin surface [127, 128]. The main endpoint in these studies is bacterial growth or bacterial competition. For example, *C. acnes* seemed to better colonize immature skin, under the differentiated epidermis. Competition was observed when *S. epidermidis* and *C. acnes* were inoculated concomitantly, with *S. epidermidis* decreasing *C. acnes* growth, while the inverse was not true [128].

Most of the published research that has used 3D models to investigate host-microbiota interactions has thus focused on the impact of individual species. Collecting bacteria from the skin, which is a very poor environment, and culturing them in conventional cell culture media probably affect their metabolism and may not reflect the natural crosstalk occurring at the skin surface.

There is a clear need to develop and improve experimental strategies for the colonization of 3D skin models with complete microbiota communities, including those directly isolated from individual humans, to more closely mimic the in vivo skin-microbiota interplay.

In 2019, Cenizo et al. [129] sampled the skin microbiota of the inner forearm of a woman and immediately inoculated the sample onto the surface of a 3D skin model. The model was followed for 7 days in culture and showed the stabilization of the number of living bacteria at a density similar to what was found on the collected skin. The microbiota-colonized model showed higher proliferation of the epidermis basal layer and increases in epidermal junctions and desquamation. This model also showed higher time stability than the same model colonized with a unique strain in which the rapid growth of *S. epidermidis* destroyed the tissue. These results suggested that the interactions occurring within diverse microbiota could prevent the outgrowth of single strains. Such models offer ways to study the impact of external factors on the composition of the skin microbiota as well as its implications for the skin response to these factors.

Even more complex models may help to establish 3D skin models as a replacement for animal models in the future. 3D skin models can include immune, nervous, pigmentation, and endothelial cells [124, 130, 131]. These models can be bioprinted to reduce their production time and improve reproducibility [132, 133] and can be inoculated with swabs collected from pathological skin lesions in which the microbiota is known to play a role (e.g*.*, atopic dermatitis, psoriasis, or acne) as well as offer new drug evaluations.

### Transplantation of skin microbiota

By analogy with fecal transplantation, which is a powerful therapeutic tool for digestive disorders [134, 135], skin microbiota transplantation studies are moving forward [136–138] and could provide a promising approach for the treatment of diseases, such as atopic dermatitis. Transplantation as a tool for correcting unsuitable armpit odor is currently a possibility under consideration. One process is focused on the removal of the malodor-causing-microbiome by the means of antibiotics, which is then replaced by a healthy nonodorous axillary microbiome. A second process consists of the application of probiotics, such as lactic bacteria or skin bacilli, but their incapacity to durably colonize the niches induced the need to find other nonodor-causing commensals that could inhibit unsuitable ones, notably *Staphylococcus hominis*, *Corynebacterium tuberculostearicum*, and *Anaerococcus* spp. [139]. This phenomenon is probably governed by more subtle principles, and it is reasonable to think that modifications may involve the microorganisms at the subtype level. The transplantation method depends on the triangle donor microbiome composition (A), the recipient microbiome composition (B), and the load of the transplant (C). Indeed, a recipient microbiome composed of *C. acnes* subtype H1 and *Leifsonia* spp. allows a better engraftment of donor strains [137]. By assessing the viability of skin microbial communities unidirectionally (from the forearm to the back of the same volunteers), Perin et al. [138] described the partial efficiency of unenriched skin microbial community transfer, but more information is needed regarding the viability of the microbes transplanted. Myles et al. pointed to *Roseomonas mucosa*, a gram-negative commensal bacterium associated with decreasing atopic dermatitis severity [136]. Nakatsuji et al. [28] successfully tested the transfer of bacteria selected for their ability to inhibit *S. aureus* in atopic dermatitis patients, which highlighted the protective effect of commensal coagulase-negative *Staphylococcus* (CoNS).

### Prebiotics and probiotics

At a time when microbiomes and their fluctuations are known to be associated with several dysfunctions, the skin microbiome is of growing interest in the field of cosmetics, focusing on the exploitation of these proprieties to improve human well-being through formulations that contain prebiotics, probiotics, or skin microbiome-friendly ingredients known as cosmeceutics.

Different firms have developed formulations containing a *Hylocereus undatus* fruit extract that can reduce the perception of skin imperfections. This extract may positively influence the skin microbiota balance, and several patents highlight this finding (Liki Von Oppen-Bezalel et al. patent n° WO2016147189A1; Korean patent n° KR20150118078A, etc.). Indeed, this plant was shown by Som et al*.* to be a major source of antioxidant substances, which are key in the skin aging mechanism [140]. Similarly, Banerjee et al. [141] controlled the formulation of an emulsion cream for topical application and considered its impact on the commensal skin flora. Bacterial extracts were also tested, such as an extract of *Shingomonas hydrophobicum* [142] and lactobionic acid from *Pseudomonas taetrolens*, for antiaging activity [143]. Other formulations containing bacterial derivative compounds (e.g., the *Lactobacillus* extract filtrate in Skinolance**®**), prebiotic peptides (ACTIBIOME™, FENSEBIOME™), or vitamins, such as niacinamide (Univerler patent WO2019086327), are provided by raw material sellers and are indicated to show prebiotic activity. Globally, this kind of information remains elusive, and firm scientific conclusions are rarely available [144].

*Lactobacillus* and *Bifidobacterium* species, which are implicated in health, were analyzed for their probiotic properties related to skin homeostasis. The tested species included *Lactobacillus reuteri* [145], *Lactobacillus acidophilus* [146], *Lactobacillus plantarum* [147–149], a formulation based on a patented *Lactobacillus* mixture (CN110121353A), *Lactobacillus helveticus* [150], *Lactobacillus rhamnosus* applied synergistically with the plant *Agastache rugosa* [151], and *Bifidobacterium breve* [152].

Recently, *Nitrosomonas eutropha,* an ammonia*-*oxidizing agent, was targeted for its antiaging properties [153, 154] and included in the Mother Dirt AO+ Mist Skin Probiotic Spray patent (JP2017519486A).

Many patents are being produced that focus on diverse bacterial strains that could improve skin well-being and the antiaging properties of cosmetics (*Streptococcus pneumoniae* and *Streptococcus thermophilus* in patents KR20180121269 and KR20180121268; the newly discovered strain *Epidermidibacterium keratini* in patent WO201804224; *Pseudoalteromomonas antartica* in patent JP2018500279A, etc.). Nevertheless, scientific data supporting their efficacy are rarely available.

## Key points and conclusions

- The skin microbiome is composed of a variety of organisms, including bacteria, archaea, fungi, and even small arthropods, which interact with each other and could be implicated in the host health status.
- The skin microbiome composition depends on many factors. These factors form an intricate network that novel sequencing technologies allow us to better understand. However, standardization of studies is required to reach strong conclusions on which innovation process could be best.
- Optimized evaluation tools, such as 3D skin models, offer ways to study the impact of modulation factors on the composition of the skin microbiota as well as its implications for the skin response.
- Presently, understanding the skin microbiome is at a turning point. The beneficial and protective role of bacterial communities in close relationship with their host is understood to be clinically manipulated (illustrated by “transplantation-like” technology) or to be an important industrial concern through the investigation of microbial-derivated products with bioactive activities.

## Data Availability

All data generated or analyzed during this study are included in this published article.

## Abbreviations

AJs: Adherent junctions
AMP: Antimicrobial peptides
CoNS: Coagulase-negative *Staphylococcus*
GNB: Gram-negative bacteria
HPV: Human papillomavirus
NGS: Next-generation sequencing
TJs: Tight junctions
